# Advances in Canine Anesthesia: Physiologically Based Pharmacokinetic Modeling for Predicting Propofol Plasma Profiles in Canines with Hepatic Impairment

**DOI:** 10.3390/ph17121720

**Published:** 2024-12-19

**Authors:** Lucas Wamser Fonseca Gonzaga, Beatriz Monte Egito, João Bosco Costa Coelho, Gabriela Pereira Souza, Frederico Severino Martins, Marcos Ferrante

**Affiliations:** 1 Department of Veterinary Medicine, Universidade Federal de Lavras, Lavras 37200-900, MG, Brazil; lucaswamserfg@gmail.com (L.W.F.G.); beatriz.egito@hotmail.com (B.M.E.); coelhojbosco@gmail.com (J.B.C.C.); mvgabrielasouza@hotmail.com (G.P.S.); 2 Department of Clinical and Toxicological Analysis, Faculty of Pharmaceutical Sciences, Universidade de São Paulo, Av. Prof. Lineu Prestes, 580, Bl. 13B, São Paulo 05508-000, SP, Brazil; fredseverinomartins@gmail.com

**Keywords:** precision anesthesia, PK model, dose individualization

## Abstract

**Background**: A PBPK model allows the prediction of the concentration of drug amounts in different tissues and organs over time and can be used to simulate and optimize different therapeutic protocols in healthy and sick individuals. The objective of this work was to create a PBPK model to predict propofol doses for healthy canines and canines with hepatic impairment. **Methods**: The study methodology was divided into two major phases, in which the first phase consisted of creating the PBPK model for healthy canines, and in the second phase, this model was adjusted for canines with hepatic impairment. The model for healthy canines presented good predictive performance, evidenced by the value of the performance measure of the geometric mean fold error that ranged from 0.8 to 1.25, meeting the double error criterion. The simulated regimen for healthy canines, i.e., of 5 mg/kg (administered as a bolus) followed by a continuous infusion at a rate of 0.13 mg/kg/min, was sufficient and ensured that all simulated subjects achieved the target plasma concentration. Canines with 60% and 40% liver function had infusion rate adjustments to ensure that individuals did not exceed the therapeutic window for maintenance of anesthesia. **Results**: The results presented in this manuscript are suggestive of the effectiveness and practicality of a PBPK model for propofol in canines, with a particular focus on hepatic impairment.

## 1. Introduction

Propofol (2,6-diisopropylphenol) is an ultra-short-acting hypnotic agent that, similar to barbiturates, induces sedation and hypnosis but lacks analgesic effects. Chemically, propofol stands out as the sole intravenous anesthetic agent suitable for the induction and maintenance of anesthesia [1].

This drug is widely employed as an intravenous anesthetic for canines, with a wide range of recommended doses for induction and maintenance of anesthesia, and is known for its short duration of action and rapid distribution and metabolism carried out predominantly by hepatic enzymes [1,2]. The most prominent undesirable effect of propofol is its marked depression of cardiovascular and respiratory parameters, which can be fatal [3].

It is imperative to note that liver dysfunctions can significantly influence the metabolism of propofol. This drug’s pharmacokinetic parameters exhibit variations between healthy canine populations and those with liver complications. In individuals with liver issues, propofol often achieves greater plasma concentrations (85%) and persists in the system for longer durations [4].

In the modern age of veterinary science, computational pharmacokinetic models have risen to prominence. These models are applied for new drug development, regulatory compliance, clinical study design, and personalized therapeutic strategies [5]. Among these, the physiology-based pharmacokinetic (PBPK) models stand out, enabling the simulations of drug concentrations over time across different tissues and organs [6,7].

PBPK modeling is a vital tool in veterinary pharmacology, providing a detailed understanding of drug kinetics in animals. This approach, which integrates animal-specific physiological and anatomical data with drug properties, is crucial for several reasons. It allows for species-specific drug dosing, enhancing the safety and effectiveness of veterinary treatments. PBPK models are instrumental in new drug development, facilitating the extrapolation of data across species. They also aid in meeting regulatory requirements for drug approval by providing a comprehensive pharmacokinetic index. Additionally, PBPK modeling holds the potential for personalized veterinary medicine, considering individual animal characteristics for tailored treatments. Overall, PBPK modeling significantly advances veterinary care by improving drug efficacy, safety, and development processes [8].

The motivation for this study lies in the existing gap in the application of physiologically based pharmacokinetic (PBPK) models for companion animals in clinical contexts. Although PBPK modeling is widely known and utilized, most published models to date focus on production animals and exhibit a relatively simplified structure [9,10]. This work represents a significant advancement in the development of a PBPK model for companion animals with a high degree of complexity, using PK-Sim (Version 11.0, 2023, www.open-systems-pharmacology.org, accessed on 10 January 2024), a robust and widely recognized software for physiologically based pharmacokinetic modeling. Unlike existing preclinical and translational studies, this model is tailored for specific clinical applications, offering novel insights and greater potential for applicability in veterinary practice. Thus, the present study contributes to filling an important gap in the field of veterinary pharmacology and expands the use of PBPK to new frontiers of application. In this study, the primary objective was to develop a physiologically based pharmacokinetic (PBPK) model specifically to predict propofol concentration in canines. Building upon this foundational model, our secondary goal was to adjust dosage regimens for simulated canine groups exhibiting varying degrees of liver impairment. This research endeavor was undertaken with the ultimate aim of enhancing the safe and efficacious administration of propofol in canines affected by hepatic conditions.

## 2. Results

The model was constructed for healthy adult canines based on the structure available in the PK-Sim software (https://www.open-systems-pharmacology.org, accessed on 10 January 2024). The structural details of the model can be found in the software’s manual. However, due to the absence of data regarding enzymatic expression and activity in canines, propofol’s total hepatic clearance was considered, resulting in 47.08 mL/kg/min.

**Figure 1:**
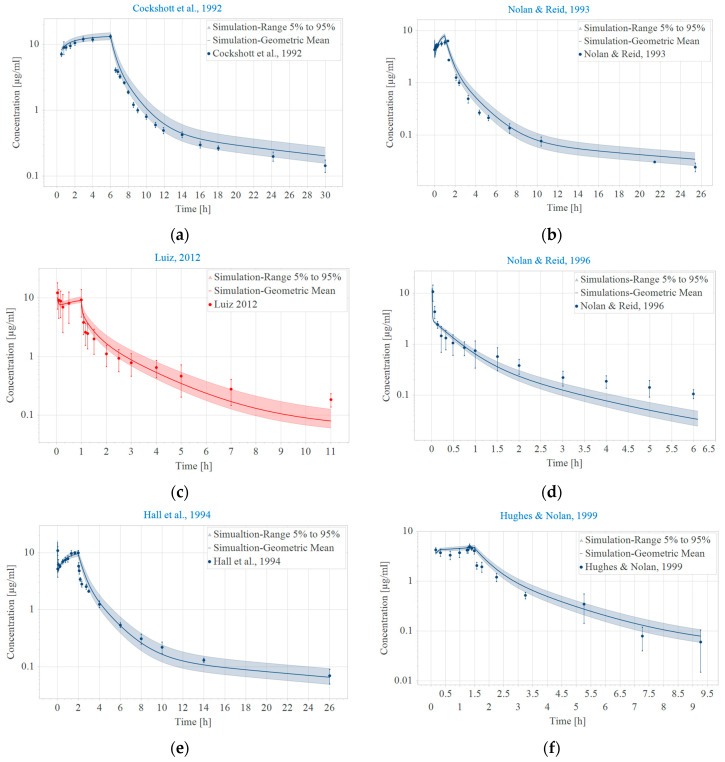

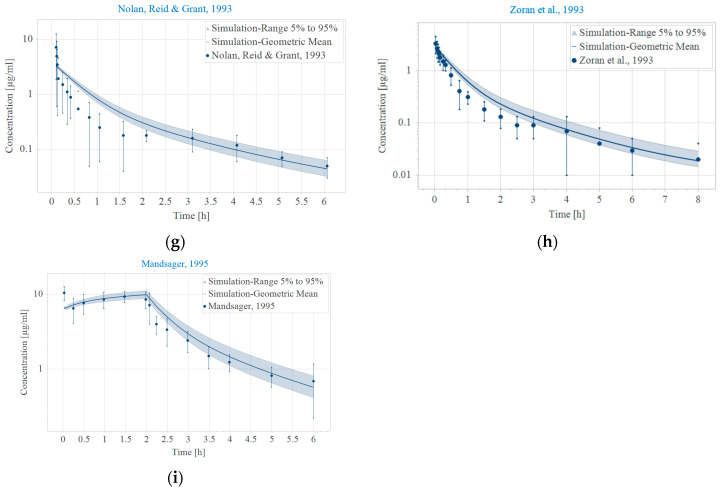
Adjustment of observed and predicted data by the pharmacokinetic model [9,10,11,12]. (**a**–**d**) for construction and [13,14,15,16,17] (**e**–**i**) for validation. Blue, venous blood; red, arterial blood. Population simulations (*n* = 1000) are shown as solid lines with shaded areas (geometric mean and geometric standard deviation). The observed data are shown in circles ± standard deviation.

**Figure 2:**
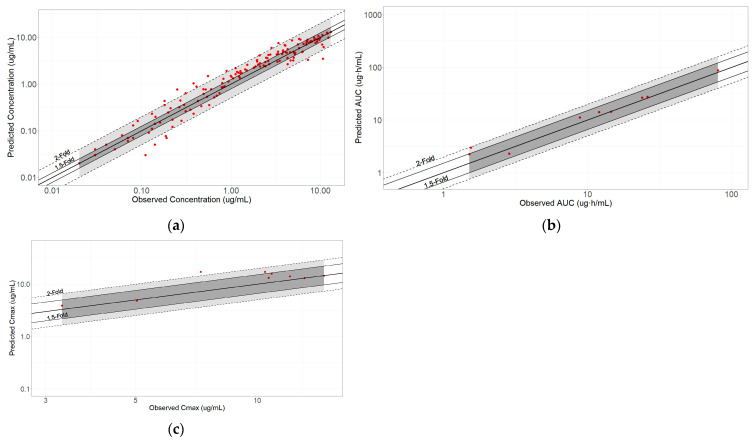
Observed and predicted values. (**a**) Plasma concentration; (**b**) area under the curve from the first to the last point; and (**c**) maximum plasma concentration.

The model was validated using data from seven studies, each employing different intravenous administration techniques—either a single bolus or a combination of bolus followed by infusion. The model effectively predicted the clinical data, as shown in Figure 1. The geometric mean fold error (GMFE) ranged between 0.8 and 1.25, which can be further explored in Figure 2 and Table 1.

**Figure 3:**
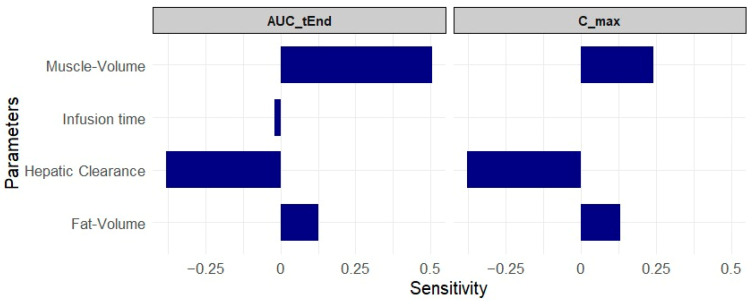
Sensitivity analysis demonstrating the impact of changes in different parameters under the model parameters.

The sensitivity analysis was conducted with all model parameters. Those with the most significant impact on AUC and maximum plasma concentration parameters are illustrated in Figure 3. A sensitivity value of +1.0 indicates that a 10% increase in the analyzed parameter leads to a 10% increase in simulated AUC, as described by Kovar et al. (2020) [20]. As illustrated in Figure 3, muscle volume was the most sensitive parameter, followed by adipose tissue volume. The remaining parameters did not exhibit an influence on the curve.

**Figure 4:**
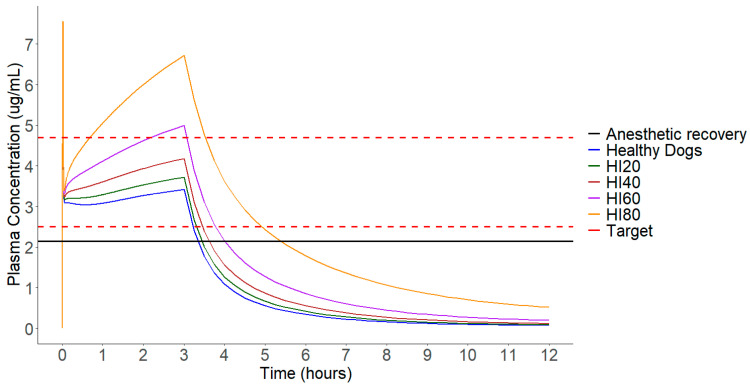
Median plasma concentration of various simulated populations 12 h after a 5 mg/kg bolus administration over 30 s followed by a 0.13 mg/kg/min infusion over 3 h. Dashed lines represent the target concentration range of 2.5 to 4.7 μg/mL. Solid black line represents the target concentration for anesthetic recovery.

**Figure 5:**
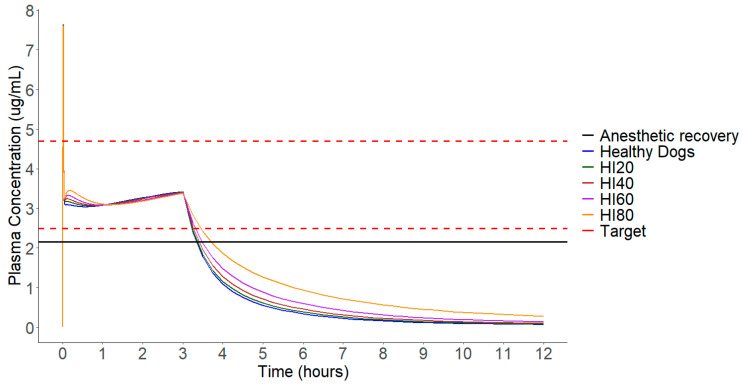
The median plasma concentration of various simulated populations 12 h after the administration of the protocol adjusted for each group. Dashed lines represent the target concentration range of 2.5 to 4.7 μg/mL. Solid black line represents the target concentration for anesthetic recovery.

Given that the concentration following the initial bolus remains unchanged with decreases in total plasma clearance (Figure 4), and noticeable modifications in plasma concentration only occur during the maintenance period, adjustments were made solely to the infusion rates. Figure 4 illustrates that the most significant difference in median plasma concentration between groups is observed at 3 h, marking the conclusion of the continuous infusion conducted for anesthetic maintenance. In the present study, simulations were conducted for individuals with decreases of 20%, 40%, 60%, and 80% in total propofol plasma clearance to mimic simulations that impair liver function. Figure 5 displays the medians of the different simulated populations, along with concentrations of 2.5–4.7 μg/mL and 2.15 μg/mL used as reference values for induction/maintenance and anesthetic recovery, respectively.

**Figure 6:**
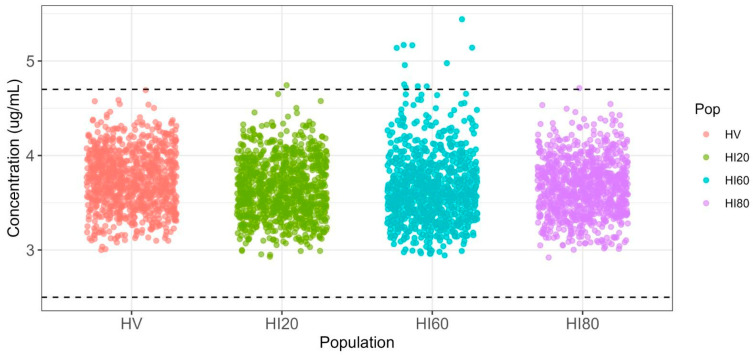
Population distribution of maximum plasma concentrations achieved after dose adjustment in canines with different levels of hepatic impairment, including healthy canines. Dashed lines represent the target concentration range of 2.5 to 4.7 μg/mL.

For healthy canines, a 5 mg/kg dose (administered as a bolus) followed by a continuous infusion at a rate of 0.13 mg/kg/min was found to be sufficient. This regimen ensures that more than 95% of 1000 simulated canines maintain their propofol concentrations within the target range at the end of the maintenance period (Figure 6).

For canines with 40% hepatic impairment, a 5 mg/kg bolus followed by a 0.105 mg/kg/min infusion was recommended. It is noteworthy that 1.5% of these canines may have plasma concentrations at the end of the maintenance period that exceed the desired upper limit, but they do not reach 6.5 μg/mL, which could lead to potential unwanted effects [21].

On the other hand, dogs with 60% hepatic dysfunction required a bolus of 5 mg/kg combined with an infusion of 0.086 mg/kg/min. Under this adjusted regimen, only 0.1% of the dogs had plasma concentrations exceeding the upper limit of 4.7 µg/mL at the end of the maintenance period.

Lastly, canines with 80% hepatic dysfunction required a 5 mg/kg bolus followed by a 0.06 mg/kg/min infusion. Additional information about this data are visualized in Figure 4, Figure 5 and Figure 6.

The recovery time increased by 27%, 68%, 172%, and 550%, compared to the healthy group (*p* < 0.0001), in the HI20, HI40, HI60, and HI80 groups, respectively, as depicted in Figure 4. These results indicate a significant relationship between anesthesia recovery time and the level of hepatic impairment.

Figure 5 shows the median concentrations of the different groups after protocol adjustments. All protocols were statistically tested based on the AUC from the start of the bolus to the end of the infusion, revealing no statistical difference between the protocols (*p* = 0.97). However, when comparing AUC3-12 h values, a significant difference between the groups is noticeable (*p* < 0.0001). Thus, even after adjusting infusion rates, differences in anesthesia recovery times among individuals still persist. Even after adjusting the doses, the recovery times vary among populations. However, when comparing the difference in recovery between the 80HI and healthy groups in the adjusted protocols (43–22 min) with the difference in the same groups without dose adjustment (143–22 min), it is noticeable that the difference is considerably smaller.

Figure 6 shows the plasma concentrations of propofol at 3 h, corresponding to the end of the continuous infusion period for anesthetic maintenance, in 1000 simulated individuals with adjusted protocols. A similar distribution of values was observed, with only a few individuals exceeding the upper limit of the anesthetic concentration window (4.7 µg/mL). No individuals surpassed the concentration of 6.5 µg/mL, which is associated with the risk of apnea [21].

## 3. Discussion

The present study’s innovative application of the pharmacokinetic model to a simulated canine population, particularly those with varying degrees of hepatic impairment. This model’s adaptability in addressing hepatic impairment is critical, as liver function significantly influences drug metabolism, especially for medications like propofol. The model provides veterinarians with detailed dosing recommendations for propofol, tailored to different levels of hepatic impairment, which range from mild (20% impairment) to severe (80% impairment). These recommendations are valuable in veterinary practice, as they allow for the customization of propofol dosages according to the individual health conditions of each patient. Such tailoring is crucial in minimizing the risk of adverse effects while ensuring that the anesthesia remains effective.

Furthermore, the results of this study emphasize the importance of maintaining propofol plasma concentrations within a specific target range. This is essential for ensuring safe anesthesia and promoting successful recovery in animals. The model’s ability to optimize dosing regimens contributes significantly to achieving these target concentrations in a high percentage of simulated canines across different groups of hepatic function. This level of precision in dosing not only improves the safety and efficacy of anesthesia but also underscores the model’s practical utility in clinical settings. It demonstrates how advanced pharmacokinetic modeling can be directly applied to enhance patient care, aligning dosing strategies with individual physiological variations. This approach, therefore, represents a significant advancement in veterinary anesthesiology, offering a more individualized and safer anesthesia management for canines with hepatic impairments.

This study utilized pharmacokinetic modeling data extracted from the literature published in journals indexed in the PubMed, Web of Science, and Scopus databases, as well as from a thesis presented by a Brazilian research team. All selected studies describe the analytical technique used and the calibration methods. The strategy of building models based on literature has been adopted in other works, such as the study conducted by Chou et al., 2022 [9], which developed three PBPK models for veterinary drugs used in production animals. We acknowledge, however, that this initial PBPK model of propofol in dogs could be improved by incorporating studies that estimate concentration profiles related to physiological variables in specific populations. In the future, we hope that new pharmacokinetic studies will be conducted to increase the complexity and predictive capacity of this PBPK model of propofol in dogs. The propofol model was developed and validated with nine different studies with varying propofol administration methods. The geometric mean fold error (GMFE) values ranging from 0.8 to 1.25 indicate that the model accurately predicts the clinical data. The acceptance criterion used was that of double error, which was also employed in the validation in the study by Kovar et al. (2020) [20]. This is a crucial step in ensuring the reliability of the model’s predictions.

The sensitivity analysis highlights the factors that influence propofol exposure in canines, including infusion rate, hepatic clearance, and the volumes of muscle and fat. This information is essential for understanding how different variables affect the drug’s pharmacokinetics, providing insights that can guide clinical practice. Propofol is a lipophilic drug with high distribution in tissues with high concentrations of fat, such as subcutaneous fat, visceral fat, and brain tissue. Obese animals can present different pharmacokinetic profiles, which makes it important to conduct future studies with this specific population [22].

Propofol has a rapid distribution leading to quick induction. It is metabolized by the enzyme CYP2B11 and has a high hepatic extraction rate. Due to these characteristics, its action is short-lived [22,23]. After bolus administration, the drug is initially distributed and subsequently eliminated, so the drug concentration post-bolus does not vary significantly with the level of hepatic impairment.

The model allowed us to highlight variations in propofol plasma concentrations during continuous infusion at a rate of 0.14 mg/kg/min, along with the corresponding adjustments for each group to prevent adverse effects. Protocol adjustments were based on the values of the area under the curve from the beginning to the end of the infusion (AUC_0–3_) (*p* < 0.05) calculated from 1000 simulated individuals. In the study conducted by Montanha et al. (2022) [24], the strategy of comparing AUC values was employed as a method to adapt dexamethasone doses in patients with liver disease and COVID-19. This approach proved effective in equalizing AUC values and consequently the drug exposure, considering that individuals exhibit changes in pharmacokinetics. In this study, the same approach was used; however, even after protocol adjustments, there was a statistical difference in post-infusion plasma concentration profiles (AUC_3–12_), suggesting a discrepancy in recovery times between individuals with hepatic impairment and healthy ones. This occurs because adjustments in continuous infusion rates alter plasma concentrations during infusion but do not alter elimination rates. Therefore, in animals with reduced hepatic function, propofol was eliminated more slowly, even after the adjustments were made.

According to Beths (2001) [21], plasma concentrations of propofol above 6.5 μg/mL in animals resulted in adverse effects such as muscle spasms and apnea. However, it is important to consider that the objective of the initial bolus is to rapidly elevate plasma concentration for a quick induction, and an initial concentration above 6.5 μg/mL for a few seconds may not necessarily be problematic [25]. On the other hand, if elevated concentrations are reached during the continuous infusion administration period, as observed in the 80HI group before adjustment, they may induce the aforementioned adverse effects [21]. Additionally, it is noted that the plasma concentration and the initial decrease in the profile of propofol after the bolus do not vary among groups with reduced total plasma hepatic clearance and the healthy group. This is because the initial plasma concentration is determined by the dose and volume of distribution, whereas the decline in initial plasma concentration is mainly due to distribution to peripheral tissues [26].

To enhance the safety of intravenous infusion anesthetic procedures in humans, infusion pumps controlled by pharmacokinetic models have been employed. For instance, Morse et al. (2021) [27] developed a specific pharmacokinetic model to guide dexmedetomidine infusions in children. Similarly, Eleveld et al. (2020) [28] adopted a similar approach to control remifentanil infusions. These models contribute to more precise and safe administration of these medications during anesthetic procedures. Edginton et al. (2006) [29] employed a physiologically-based pharmacokinetic (PBPK) approach to develop a model aimed at controlling propofol infusions in humans, demonstrating the feasibility of using physiological modeling for this purpose. In veterinary medicine, Cattai et al. (2019) [30] proposed a three-compartment pharmacokinetic model for application in target-controlled infusion systems. This approach allowed infusions to achieve different target plasma concentrations with acceptable accuracy. However, the model has limitations as it does not allow adjustments based on the patient’s metabolic capacity, which, as evidenced in this study, is crucial for prolonged propofol infusions. Therefore, the model developed in this study would be highly useful for use in target-controlled infusion systems in patients with hepatic insufficiency requiring total intravenous anesthesia procedures.

The software PK-SIM allows for the construction of PBPK models capable of estimating drug concentrations in various compartments of body tissues, such as the central nervous system’s interstitial space. This ability to estimate concentrations in specific locations proves highly useful for inferring the therapeutic effects achieved in protocol simulations. However, to accurately estimate concentrations in peripheral compartments, it is necessary to include observed concentrations in the peripheral tissues in the model’s construction and validation. Luo et al. (2004) [31] quantified propofol concentrations in the plasma and cerebrospinal fluid of canines that could be used for model construction. However, this study did not report details about the administration protocols used, which hindered the accurate estimation of propofol concentration in the cerebrospinal fluid. It is hoped that in the future, studies will be conducted with sufficient details so that the current model can be complemented with the capability to predict cerebrospinal fluid concentration accurately. This would enable a more precise prediction of propofol’s anesthetic effects in target-controlled infusion systems.

Precision medicine is an approach that considers interindividual variability [32]. In the case of antimicrobial protocols, this approach can be implemented through pharmacokinetic and/or pharmacodynamic models that include covariates of parameters. For instance, the model proposed in Tod et al. (2000) [33] allows for dose adjustments of isepamicin based on creatinine renal clearance. In the present study’s model, the proposed covariate is the degree of hepatic impairment. Therefore, for its application in anesthetic routine, it is essential to determine biomarkers that enable the estimation of hepatic dysfunction level.

The liver is the main organ responsible for metabolism and has a significant ability to withstand damage, which makes estimating its level of function challenging [34]. Traditionally, liver injuries are assessed in clinical practice through enzymatic profiles; however, these parameters indicate cellular damage but are not reliable indicators of functionality. In this case, measuring bile acids and ammonia is more appropriate [34]. Hill et al. (2011) [35] discovered a positive correlation between bile acid levels and liver function in canines. However, these biomarkers did not show acceptable sensitivity and specificity for categorizing levels of hepatic dysfunction [35].

Recent studies based on metabolomic analysis in a porcine model managed to estimate degrees of hepatic dysfunction through a multiple regression model from the quantification of five metabolites (gamma-aminobutyric acid, methionine, glucose, malic acid, and tryptophan) [36]. Therefore, using these metabolites as biomarkers to characterize covariates for therapeutic protocol adjustments is a promising strategy. Another feasible alternative would be to estimate the degree of hepatic dysfunction by determining the plasma concentration of a known drug at a predetermined time; elevated plasma concentration values would indicate lower hepatic metabolism capacity. This approach is known as the ‘single point sample’ method and was used in Yang et al. (2018) [37], where they quantified plasma concentrations of midazolam four hours after administering a 2 mg dose in humans to estimate the degree of hepatic dysfunction. This alternative presents an advantage as midazolam and propofol are metabolized by the enzyme CYP2B11 [38], and midazolam is widely used in veterinary clinical practice.

Although many complications occur during anesthesia, 47% to 60% of anesthetic-related deaths of canines and cats occur during the postoperative anesthesia period. Thus, the care and continuous monitoring of patients in anesthetic recovery are as important as vigilance during anesthesia maintenance, so that an optimal anesthetic recovery time generally ranges from 10 to 30 min after the end of anesthesia [2]. Therefore, reducing the difference in recovery time from 6-fold (from 22 min to 143 min) to 2-fold (from 22 min to 43 min) between the healthy group and the 80HI could have a significant impact on mortality rates associated with the anesthetic recovery period.

The assessment of a successful anesthetic protocol significantly depends on the patient’s recovery, which can be affected by the concurrent administration of other drugs. Therefore, it is crucial to emphasize that in multimodal anesthetic protocols, the pharmacological effects of plasma concentrations are altered. Thus, using the plasma concentration value of propofol to estimate recovery time, as determined in Beths et al. (2001) [21], should only be considered as a relative comparison strategy between healthy patients and those with hepatic dysfunction. On the other hand, another limitation of this study is that it does not allow an estimation of the impact on the pharmacokinetic profile of propofol from the concurrent administration of other widely used anesthetic co-adjuvants. However, this model will serve as a basis for future studies of drug–drug interactions (DDI).

Overall, the results presented in this manuscript have significant clinical implications for veterinary medicine. They provide veterinarians with a tool to enhance the precision of propofol administration in canines, especially those with liver conditions. This contributes to safer anesthesia procedures, reduces potential risks, and ultimately leads to better clinical outcomes. The manuscript’s findings elevate the standards of veterinary practice and underscore the importance of individualized therapeutic strategies.

While this manuscript provides valuable insights, it is important to note that the model’s predictions may benefit from further refinement and validation in real clinical settings. Additionally, future research could explore the applicability of the PBPK model to other drugs and species, broadening its impact on veterinary pharmacology.

One of the biggest challenges in the undergraduate teaching of veterinary anesthesiology/pharmacology is for future professionals to comprehend the inter- and intra-species differences, including those existing within and between populations [39]. Consequently, training veterinary anesthetists capable of adapting protocols to different needs requires many years of education [40]. In this sense, the use of models/apps in education allows students to grasp cause-and-effect relationships more efficiently [41]. Therefore, it is expected that the model developed in this study can contribute to improving the training of future veterinary anesthetists.

## 4. Materials and Methods

### 4.1. Software

The PBPK model, which is based on physiological processes, was created using the PK-Sim^®^ modeling software (Version 11.0, 2023, www.open-systems-pharmacology.org, accessed on 10 January 2024). The data used in this model were sourced from published clinical studies. To digitize these data, we used WebPlotDigitizer (Version 4.8, 2024, https://automeris.io/WebPlotDigitizer, accessed on 10 January 2024).

In terms of analysis, all the pharmacokinetic parameters and the measures of how well the model performed were derived from both simulated and actual (observed) data. These analyses were performed using R, a statistical computing software. Specifically, we used version 3.6.1 of R.

### 4.2. Data

The pharmacokinetic profiles observed in vivo were obtained from the scientific literature. Comprehensive searches were conducted in databases such as PubMed, Web of Science, and Scopus, focusing on studies reporting plasma concentration of propofol over time in healthy dogs. As a result, 9 clinical datasets from 9 studies (listed in Table 2) were identified and used for the development and validation of the model.

### 4.3. Model Development

In our study, the parameters specific to propofol were gathered from a variety of different sources. These sources and the respective parameters are comprehensively listed in Table 3. Key attributes of propofol, such as its molecular weight (MW), acid dissociation constant (pKa), and the proportion of the drug that remains unbound in the plasma, were obtained from the PubChem database. Canines were simulated in PK-Sim to reproduce the characteristics of the animals in the studies, based on weight, using the physiological and anatomical parameters provided directly by the software.

Furthermore, the partition coefficients were determined using the “PK-Sim-standardized” method. This method is provided by the PK-Sim modeling.

Additionally, the unspecific hepatic clearance rate of propofol was also calculated. For this calculation, we again employed the standard method provided by PK-Sim.

The comprehensive PBPK prediction approach was segmented into a three-stage method, as previously delineated by Martins et al. (2023) [5] and Martins et al. (2021) [42], with modifications. During the first stage, development and performance verification centered exclusively on studies involving intravenous (i.v.) bolus administration. The subsequent stage incorporated only those studies that utilized infusion. The final stage was dedicated to simulating a population with hepatic impairment (HI).

### 4.4. Model Performance Verification

The exposure parameters generated by the PBPK model were validated using available pharmacokinetic data for propofol. The performance verification involved the simulations of 1000 subjects, incorporating consistent demographic and dosing details from the literature. This considered factors such as dose, route of administration, age, sex distribution (male/female ratio), and body weight. The overall accuracy of the predicted pharmacokinetic parameters was evaluated using the geometric mean fold error (GMFE) of the AUClast as follows (Equation (1)):

(1) $$GMFE=10^{X},\;with\;x=\frac{1}{n}\sum_{i=1}^{n}\left|{log}_{10}\left(\frac{{A\hat{U}C}_{i}}{{AUC}_{i}}\right)\right|$$

where *AÛC_i_* represents the area under the curve of the plasma concentration profiles predicted by the model and *AUC_i_* represents the area under the curve of the observed plasma concentration profiles.

### 4.5. Model Application

After model validation, a virtual population of dogs with hepatic impairment (HI) was generated. Each population subgroup consisted of 1000 individuals, with body weight ranging from 7.5 to 13.5 kg, uniformly distributed, as well as the physiological and anatomical parameters. Hepatic impairment was simulated at four levels: 20%, 40%, 60%, and 80%. The safety and efficacy of maintaining anesthesia and facilitating animal recovery were based on a study reported by Beths et al. (2001) [21]. For induction and maintenance, it was determined that a dosage range of 5 mg/kg over 60 s (target plasma concentration of 3.0 µg/mL) followed by a 3-h infusion (with target plasma concentrations of 2.5 and 4.7 µg/mL) should be used.

### 4.6. Dosage Adjustment for Individuals with Hepatic Impairment

To adjust the dosage for canines with hepatic impairment, we reduced the infusion rate. The protocols were designed taking into account no statistical difference (*p* > 0.05) in the area under the curve (AUC0-3h) between the groups. We conducted a statistical test on the AUC3-12h values when adjusting the protocols to evaluate the difference in recovery. This adjustment included performing a Kolmogorov–Smirnov test to verify if the data followed a normal distribution. Subsequently, a Kruskal–Wallis test was conducted to assess differences between the groups, followed by a Dunn’s test for multiple comparisons.

## 5. Conclusions

In conclusion, the results of this study show the effectiveness and practicality of a PBPK model for propofol in canines, with a particular focus on hepatic impairment. The model allowed the determination of the doses used in the anesthetic protocols of propofol in canines with hepatic impairment with adequate precision, thus allowing individuals to avoid reaching plasma concentrations associated with a risk of adverse effects and excessively prolonged recovery periods. This research advances our understanding of propofol pharmacokinetics in veterinary medicine and has the potential to improve the safety and efficacy of anesthesia in canines, ultimately benefiting both veterinarians and their canine patients.

## Figures and Tables

**Table 1:** Geometric mean fold error (GMFE) of AUC.

Reference	AUClast		
	Pred. (µg·h/mL)	Obs. (µg·h/mL)	Pred./Obs.
Reid and Nolan (1996) [11]	2.25	2.86	0.79
Nolan and Reid (1993) [12]	13.81	12.04	1.15
Luiz (2012) [13]	14.05	14.61	0.96
Cockshott et al. (1992) [14]	85.63	80.53	1.06
Hall et al. (1994) [15]	26.93	26.07	1.03
Hughes and Nolan (1999) [16]	10.92	8.87	1.23
Nolan et al. (1993) [17]	2.92	1.56	1.87
Zoran et al. (1993) [18]	2.19	1.51	1.45
Mandsager et al. (1995) [19]	25.89	23.93	1.08

**Table 2:** Overview of clinical studies used to build and evaluate the propofol PBPK model.

Paper	Dose_a_ (mg/kg)	Dose_b_ (mg/kg/min)	Administration Route	Body Weight (kg)	Model Application	*n* and Breed
Cockshott et al., 1992 [14]	7	0.47	Bolus + Infusion (6 h)	17.9 ± 0.3	Development	3; beagle
Nolan and Reid, 1993 [12]	4	0.4	Bolus + Infusion (1 h)	21.25	Development	7; beagle
Luiz, 2012 [13]	8	0.4	Bolus + Infusion (1 h)	10.7 ± 1.5	Development	6; mixed breeds
Reid and Nolan, 1996 [11]	5		Bolus	29 ± 7.4	Development	6; varied breeds
Hall et al., 1994 [15]	6.9	0.4	Bolus + Infusion (2 h)	21.5	Validation	6; mixed breeds
Hughes and Nolan, 1999 [16]	4	0.3 (opening) and 0.2 (last)	Bolus + Infusion (opening 20 min; last 70 min)	25.58 ± 3.38	Validation	8; greyhound
Nolan, Reid, and Grant, 1993 [17]	6.5		Bolus	29.5	Validation	6; varied breeds
Zoran et al., 1993 [18]	5		Bolus	32.7	Validation	10; greyhound
Mandsager, 1995 [19]	10	0.4	Bolus + Infusion (2 h)	25.5 ± 4.5	Validation	5; greyhound

**Table 3:** Substance characteristics and drug-dependent parameters used in constructing the PBPK model.

Parameters	Value	Unit	Source	Reference	Literature Values	Description
Physico-Chemical Characteristics						
MW	178.27	g/mol	lit.	PubChem, CID 4943	178.27	Molecular weight
pKa (acid)	11.1		lit.	PubChem, CID 4943	11.1	Acid dissociation constant
logP	3.49		optim.	PubChem, CID 4943	3.79	Lipophilicity
Sol_pH_	0.12	mg/mL	lit.	PubChem, CID 4943	0.12	Solubility at reference pH
Distribution						
B:P ratio	2.36		optim.			Blood/Plasma concentration ratio
f_u_	2	%	optim.	Cockshott et al., 1992 [14]	1.7 ± 0.08	Unbound fraction
Elimination						
Unspecific hepatic clearance	47.08	mL/min/kg	optim.	[11,12,13,14,15,16,17,18,19]	34.0 ± 1; 34.5 ± 12.1; 50.1 ± 3.9; 81.95 ± 26.98	Elimination from plasma

## Data Availability

Data are contained within the article.
